# Functional Variability and Interchangeability of Commercial Limestone Powders in Concrete: An Experimental and Multivariate Screening Approach

**DOI:** 10.3390/ma19194133

**Published:** 2026-09-28

**Authors:** Izabela Skrzypczak, Agnieszka Leśniak, Marta Kiernia-Hnat, Maciej Barć, Łukasz Kosturkiewicz, Marta Słowik

**Affiliations:** 1 Faculty of Civil and Environmental Engineering and Architecture, Rzeszow University of Technology, 35-029 Rzeszow, Poland; izas@prz.edu.pl; 2 Faculty of Civil Engineering, Cracow University of Technology, 31-155 Krakow, Poland; 3 Construction Technology Centre Institute for Testing and Certification Ltd., 35-105 Rzeszow, Poland; m.kiernia@ctb-ibc.pl (M.K.-H.); m.barc@ctb-ibc.pl (M.B.); 4 Nordkalk Wapno Sp. z o.o., 26-052 Nowiny, Poland; lukasz.kosturkiewicz@nordkalk.com; 5 Faculty of Civil Engineering and Architecture, Lublin University of Technology, 20-618 Lublin, Poland; m.slowik@pollub.pl

**Keywords:** limestone powder, concrete, material variability, functional interchangeability, multivariate analysis, principal component analysis, hierarchical clustering

## Abstract

**Highlights:**

- Commercial limestone powders showed markedly different concrete response profiles.
- Powder performance varied with concrete class and fly-ash replacement level.
- PCA explained 81.26% of the total response variance in the first two components.
- Ward clustering revealed descriptive groups of powders with similar multivariable response profiles.
- The results show that commercial limestone powders should not be assumed to be functionally interchangeable.

**Abstract:**

Commercial limestone powders marketed under the same general material designation may differ in composition and particle characteristics, raising questions about their functional interchangeability in concrete. This study combined experimental concrete testing with multivariate data analysis to compare six commercially available limestone powders in C12/15 and C20/25 concretes, with 50% or 100% replacement of the original fly-ash fraction. Slump, air content, 7- and 28-day compressive strength, and water penetration were evaluated relative to class-specific reference concretes. Robust screening, rank-based correlation, principal component analysis (PCA), and hierarchical clustering were used as exploratory tools. Individual 28-day strength-retention ratios ranged from 0.501 to 1.094, while water-penetration performance showed a different, matrix-dependent ranking. The first two principal components explained 81.26% of the total response variance. Ward clustering produced a descriptive two-group structure, C–D–E and A–B–F, with Powder F appearing separate in a three-cluster interpretation. Because each powder–class–replacement combination was represented by a single batch and the 24 observations were repeated evaluations of six powders under four related conditions, the results are interpreted as exploratory. Within the investigated conditions, commercial limestone powders should not be assumed to be functionally interchangeable, and qualification should consider multivariable response profiles in the target concrete matrix.

## 1. Introduction

The cement and concrete industries are under increasing pressure to reduce their environmental impact while maintaining the mechanical performance, durability, availability, and economic competitiveness expected of construction materials. Cement manufacture remains an important source of anthropogenic CO_2_ emissions because emissions arise both from the energy required for clinker production and, more fundamentally, from the calcination of carbonate raw materials [1,2,3]. Current decarbonisation strategies therefore extend beyond improvements in kiln efficiency and fuel substitution and increasingly emphasise lower clinker factors, more efficient use of Portland cement in concrete, and wider deployment of supplementary cementitious materials (SCMs) and other mineral constituents [2,3,4,5,6,7]. Recent assessments of the cement and concrete sectors confirm that material substitution and binder optimisation remain essential components of near- and medium-term decarbonisation pathways [2,3].

Siliceous fly ash, ground granulated blast-furnace slag, silica fume, and other industrial mineral additions have been used for decades to modify cementitious systems. Depending on their chemical and mineralogical composition, such materials may contribute through pozzolanic or latent hydraulic reactions and can improve selected mechanical, transport, and durability-related properties of concrete [6,7,8,9,10]. Their future availability, however, cannot be assumed to be unlimited. The progressive transformation of the energy sector is reducing the generation of coal-combustion fly ash, while the availability of high-quality blast-furnace slag may become more constrained as steelmaking technologies evolve [4,7,8]. Recent reviews of SCMs have therefore placed increasing emphasis on alternative sources, material characterisation, and performance-based qualification rather than on material designation alone [7,11]. Moreover, industrial by-products and naturally derived mineral additions can exhibit substantial variability in fineness, mineralogy, glass content, loss on ignition, chemical composition, and reactivity. Consequently, the development of lower-clinker concrete requires not only access to alternative mineral materials but also robust procedures for determining whether individual commercial products are suitable for a particular cementitious system [7,8,9,10,11].

Limestone powder is particularly relevant in this context. Limestone is widely available, can be processed using established crushing and grinding technologies, and has a long history of use as a constituent of Portland-limestone and blended cements. Its role in cementitious systems is considerably more complex than that of a purely inert volumetric filler. Four principal contributions are commonly distinguished: filler, nucleation, dilution, and chemical effects [12,13,14,15]. Fine limestone particles can occupy spaces between larger cement grains, modify particle packing, and influence capillary pore development. Their surfaces may also provide nucleation sites for hydration products and thereby accelerate early hydration. Conversely, replacement of a reactive constituent by limestone decreases the amount of potentially reactive material and may cause a dilution effect when the replacement level becomes excessive [12,13,14,15]. Calcium carbonate may additionally interact with aluminate-bearing phases, modifying AFm/AFt equilibria and promoting the formation of hemi- and monocarboaluminate phases [15,16,17,18]. Recent studies on micro- and nano-limestone confirm that these mechanisms remain strongly dependent on particle size and that the balance between nucleation, dilution, and chemical effects changes with limestone fineness [19].

The interaction between limestone powder and fly ash is especially important because these materials may perform different but complementary functions. Fly ash supplies aluminosilicate phases capable of pozzolanic reaction, particularly at later ages, whereas limestone can affect early hydration, particle packing, and carbonate–aluminate reactions. De Weerdt et al. demonstrated that limestone in fly-ash-containing cement systems can stabilise ettringite and promote carboaluminate formation, thereby changing hydrate assemblages and the volume of solid hydration products [17,18]. These findings established an important mechanistic basis for ternary Portland cement–fly ash–limestone systems. More recent studies and reviews continue to identify limestone–SCM synergy as a promising route for increasing clinker substitution, while simultaneously emphasising that the magnitude of the effect depends on SCM reactivity, reactive alumina content, limestone fineness, sulphate balance, replacement level, and curing conditions [20,21]. Thus, limestone should not be considered a universally equivalent replacement material; its performance must be assessed within the specific binder and concrete matrix in which it is used.

Particle characteristics are among the most important sources of variability in limestone powders. Particle-size distribution, specific surface area, particle shape, and the proportion of ultrafine particles affect water demand, rheology, packing density, hydration kinetics, and the extent of the nucleation effect [12,13,14,19,22,23,24,25]. Very fine limestone can accelerate hydration and improve packing, whereas excessive fineness may increase water demand or plastic viscosity and alter the effectiveness of chemical admixtures. Coarser limestone particles may contribute less to nucleation and micro-filler effects and behave predominantly as solid replacement. Recent evidence indicates that the effect of limestone on hydration kinetics changes markedly with particle size, with finer powders generally exhibiting stronger nucleation-related effects than coarser materials [19]. Accordingly, concrete response cannot be predicted from limestone dosage alone; fineness and particle-size distribution must be considered together with the characteristics of the cementitious system.

The physicochemical characteristics of limestone-derived products introduce an additional source of variability. Materials marketed under the generic designation of “limestone powder” may contain different proportions of calcite, dolomite, quartz, clay minerals, and other minor constituents, depending on geological origin and processing history. Even products satisfying comparable grading requirements may therefore differ in surface properties, morphology, hardness, carbonate composition, and interactions with cement or admixtures. In addition, limestone should not automatically be treated as fully inert. The classical work in [15] demonstrated the influence of calcium carbonate on aluminate-bearing hydrates, while later studies confirmed that limestone can modify hydration kinetics and phase assemblages, particularly in systems containing reactive alumina [16,17,18,20,21]. This is important from the perspective of commercial material selection because nominal product designation or carbonate content alone may not adequately predict concrete performance.

The engineering consequences of these material-dependent effects have been reported for both fresh and hardened concrete. Limestone powder may influence consistency, yield behaviour, plastic viscosity, air content, setting, early-age and later-age strength, permeability, shrinkage, and other durability-related properties [12,13,22,26,27,28]. However, the direction and magnitude of the response are not universal. Beneficial effects are frequently observed at moderate replacement levels or where particle packing and early hydration are improved, whereas excessive replacement of reactive constituents can reduce later-age strength and unfavourably alter transport properties because of dilution [13,22,26,27]. Comparative studies of limestone fillers and reactive mineral additions indicate that their effects cannot be regarded as equivalent over the entire replacement range. Limestone may contribute favourably through physical and early-age effects, whereas pozzolanic materials can provide an additional contribution to later-age strength and durability through continued reaction [26,29]. A recent review of SCM performance likewise stresses that replacement level, particle characteristics, and material origin are decisive for the balance between workability, mechanical performance, and durability [11].

These considerations become particularly important when limestone powders are commercially available products manufactured primarily for sectors other than cement or concrete. Powders used in the glass, paper, chemical, agricultural, metallurgical, or other industries may be readily available at industrial scale, but their suitability for concrete cannot be inferred from their commercial designation. From the perspective of a concrete producer, locally available powders may originate from different deposits and production routes and may therefore exhibit different particle-size distributions and physicochemical characteristics. Recent comparative research has begun to confirm this practical concern: Safiddine et al. compared three limestone fillers of different origins and processing routes and reported substantially different rheological and mechanical responses in cement mortars [30]. Nevertheless, studies of this type remain limited in the number of commercial materials and performance criteria considered. A laboratory investigation based on a single, highly characterised model limestone therefore cannot fully represent the practical material-selection problem encountered in concrete production.

This need is reinforced by the multidimensional nature of concrete performance. An assessment based solely on one mean response, most commonly compressive strength, can conceal important trade-offs. A limestone powder associated with relatively high compressive strength may simultaneously increase air content, alter workability, or lead to less favourable water-penetration results. Conversely, a material with only moderate mechanical performance may provide a more balanced overall response. For screening purposes, the problem is therefore not limited to determining which powder gives the highest value of one property, but extends to identifying response patterns across several mutually relevant performance indicators.

Multivariate data-analysis techniques provide a useful framework for this type of problem. Principal component analysis (PCA) can reduce the dimensionality of correlated performance data and identify the variables primarily responsible for differentiation between mixtures, whereas hierarchical clustering can reveal groups of materials with similar multivariable response profiles [31,32,33]. Correlation analysis and normalised performance measures can further support interpretation of relationships between fresh and hardened properties. PCA has already been shown to be useful in concrete mixture analysis by identifying relationships between mixture composition and multiple concrete properties and by simplifying interpretation of complex experimental datasets [34]. In the present context, the value of multivariate analysis lies not in replacing mechanistic material characterisation, but in providing an objective screening framework for comparing several candidate mineral powders on the basis of their observed behaviour in concrete.

A performance-based multivariate approach is especially appropriate for commercial limestone powders because the practical question is not simply whether one mixture is stronger than another. More importantly, it is necessary to determine whether powders marketed under the same generic material category can be regarded as interchangeable, within a defined application, whether individual products produce consistently atypical responses, and whether materials exhibiting similar overall behaviour can be grouped descriptively from their multivariable response profiles. From this perspective, material-to-material variability is itself a relevant engineering characteristic. If nominally similar limestone powders lead to markedly different workability, air content, strength development, or water penetration under identical mixture conditions, product-specific verification becomes necessary before substitution can be recommended. 

The literature has established a broad understanding of limestone mechanisms, including the effects of dosage, fineness, limestone–fly-ash interactions, and the behaviour of binary and ternary cementitious systems [12,13,14,15,16,17,18,19,20,21,22,23,24,25,26,27,28,29]. Recent studies have also begun to compare limestone fillers of different origins [30]. However, these investigations generally involve a limited number of materials or focus on selected responses, particularly rheological or mechanical properties. Systematic studies involving several commercially available limestone powders tested under identical concrete conditions and evaluated simultaneously across fresh, mechanical, and transport-related performance indicators remain scarce. This distinction is important because industrial material selection concerns actual market products rather than an idealised limestone of controlled origin. The novelty of the present study therefore lies not in demonstrating that limestone powder can be used in concrete, but in examining whether multiple commercial limestone powders can be considered functionally interchangeable when assessed across several fresh- and hardened-concrete properties and in integrating experimental concrete testing with a multivariate, performance-based screening procedure to distinguish their response profiles. (Figure 1).

Accordingly, the objective of this study was to combine experimental concrete testing with multivariate data analysis to evaluate the performance variability and functional interchangeability of six commercially available limestone powders. The powders were investigated under identical mixture conditions using two concrete strength classes and two fly-ash replacement levels. Consistency, air content, 7-day and 28-day compressive strength, and water penetration were treated as complementary performance indicators. Statistical screening, normalised performance evaluation, correlation analysis, and exploratory multivariate grouping were used to identify systematic response patterns and to determine whether the tested commercial powders could reasonably be regarded as interchangeable within the investigated conditions. The proposed approach is therefore a performance-based screening framework, not a universal acceptance procedure, and its conclusions are limited to the concrete matrices, replacement levels, curing ages, and properties examined in this study.

## 2. Materials and Methods

### 2.1. Concept of the Experimental Programme

The experimental programme was carried out using two ordinary concrete mix designs representative of strength classes C12/15 and C20/25. Both reference concretes contained Portland cement CEM I 42.5 R, siliceous fly ash, natural aggregates, potable tap water, and a plasticising admixture. The cement used in both concretes was CEM I 42.5 R produced by Cement Ożarów, while the fly ash originated from the Połaniec power plant. The aggregate system consisted of natural 0/2 mm sand from Czarna Sędziszowska and 2/8 mm and 8/16 mm gravel fractions supplied by Kruszgeo. Plast 317 (Chryso Polska Sp. z o.o., Warsaw, Poland) was used as the plasticising admixture. The same cement, aggregate fractions, water source, and admixture type were maintained within each concrete class so that the effect of replacing fly ash with limestone powder could be assessed without simultaneous changes in the remaining mixture constituents.

Six commercial limestone powders were investigated. The materials were market-available products used primarily in industrial applications other than concrete production. They were supplied in anonymised form by the Polish Lime Association, an industry organisation associating Polish lime producers, on behalf of its member companies. Throughout the experimental programme the materials were tested exclusively under codes A–F; their producer and trade identity were disclosed neither to the laboratory staff performing the measurements nor to the academic research team, and remain unknown to the authors. This procedure was adopted in order to eliminate supplier-related bias in the experimental evaluation and in the interpretation of the results. In the manuscript, the powders are treated as six distinct commercial products and compared under identical concrete mixture conditions. The assessment was performance-based and focused on their effects on fresh- and hardened-concrete properties rather than on an a priori ranking derived from chemical composition or a single physical parameter.

To document the material variability among the investigated commercial products, selected chemical, mineralogical, and physical characteristics of the six limestone powders are summarised in Table 1. The reported parameters provide a basic description of the powders and demonstrate differences in composition and particle-related properties. The characterisation was performed at the Construction Technology Centre—Institute for Testing and Certification Ltd. and, for selected determinations, at the laboratory of Cement Ożarów S.A. According to the laboratory characterisation records, oxide composition was determined by X-ray fluorescence (XRF), calcite content by X-ray diffraction (XRD), specific surface area by the Blaine air-permeability method, and particle size using the HELOS particle-size analysis system. Density values were taken from the archived laboratory characterisation records; however, the exact density determination procedure could not be verified. The dataset available for the present manuscript did not retain the complete standard identifiers for all characterisation procedures; therefore, unverified standard numbers have not been assigned. These descriptors were used to contextualise the concrete responses, whereas the primary screening and multivariate analyses were based on measured fresh- and hardened-concrete performance.

### 2.2. Concrete Mixture Design and Experimental Matrix

Two reference concrete compositions were used. The C12/15 reference concrete contained 210 kg/m^3^ of CEM I 42.5 R, 100 kg/m^3^ of fly ash, 220 kg/m^3^ of water, 720 kg/m^3^ of 0/2 mm aggregate, 400 kg/m^3^ of 2/8 mm aggregate, 700 kg/m^3^ of 8/16 mm aggregate, and 2.1 kg/m^3^ of Plast 317. The C20/25 reference concrete contained 280 kg/m^3^ of CEM I 42.5 R, 100 kg/m^3^ of fly ash, 225 kg/m^3^ of water, 655 kg/m^3^ of 0/2 mm aggregate, 400 kg/m^3^ of 2/8 mm aggregate, 700 kg/m^3^ of 8/16 mm aggregate, and 2.8 kg/m^3^ of Plast 317. These compositions are summarised in Table 2.

For each limestone powder, fly ash was replaced on an equal-mass basis at two levels: 50% and 100% of the fly ash content of the corresponding reference concrete. Thus, the 50% replacement variants contained 50 kg/m^3^ of fly ash and 50 kg/m^3^ of limestone powder, whereas the 100% replacement variants contained 100 kg/m^3^ of limestone powder and no fly ash. Cement content, water content, aggregate proportions, and admixture dosage were kept constant within each strength class. The experimental structure comprised six powders × two concrete strength classes × two fly-ash replacement levels, giving 24 limestone-containing concrete variants, together with two class-specific reference concretes. Because substitution was performed by mass rather than by equal solid volume, differences in powder density and in the unknown fly-ash density could modify the total solid volume and packing. This factor is inseparable from the product-specific response in the present dataset and should be controlled explicitly in future replicated experiments.

The two replacement levels were deliberately chosen to represent two distinctly different technological situations. The 50% level retained half of the original fly ash and therefore represented a hybrid fly ash–limestone system, whereas the 100% level represented complete substitution of the fly ash by limestone powder. This contrast allowed the assessment to distinguish powders that maintained acceptable performance only in the presence of residual fly ash from powders showing stable behaviour after complete fly-ash replacement.

### 2.3. Mixing, Specimen Preparation and Test Methods

Laboratory batches were produced from previously weighed constituents and mixed until a visually homogeneous mixture without signs of segregation was obtained. After fresh-concrete testing, specimens were cast in cubic moulds and compacted in two layers using a vibrating table. The specimens were demoulded on the following day and cured under laboratory-controlled conditions in accordance with EN 12390-2 [35]. The test programme included 100 × 100 × 100 mm cubic specimens for compressive-strength testing and 150 × 150 × 150 mm cubic specimens for water-penetration testing.

Fresh-concrete consistency was determined by the slump test in accordance with EN 12350-2 [36]. Air content was determined using the pressure method according to EN 12350-7 [37]. These two measurements were performed at the batch level and were therefore treated as technological characteristics of each concrete mixture rather than as replicated specimen-level observations.

Compressive strength was determined after 7 and 28 days in accordance with EN 12390-3 [38]. For each age and mixture, three individual compressive-strength results were available. These three specimens were obtained from the same concrete batch and therefore constitute technical, within-batch replicates rather than independent experimental replicates. Their arithmetic mean was used as the batch-level response in the multivariate analysis, while specimen-level standard deviation and coefficient of variation were retained only as measures of test dispersion.

Resistance to water penetration was evaluated after 28 days in accordance with EN 12390-8 [39]. Three 150 × 150 × 150 mm specimens from the same batch were tested for each mixture, and the mean maximum depth of water penetration was adopted as the batch-level response. As with compressive strength, these three measurements represent technical replication within a batch, not independent batch replication. Lower penetration depth was interpreted as more favourable performance. Slump and air content were measured once per batch, whereas compressive strength and water penetration were represented by the mean of three within-batch specimens. Complete individual specimen results are provided in Supplementary File S1.

### 2.4. Data Structure and Statistical Screening

The statistical procedure was designed specifically for the structure of the available experimental data. Each powder–concrete-class–replacement-level combination corresponded to one laboratory batch. Consequently, the individual strength and water-penetration specimens represented within-batch technical replicates and were not treated as independent experimental replicates. The study therefore does not quantify batch-to-batch variability associated with day-to-day mixing, aggregate moisture, or other production fluctuations. This limitation affects the strength of inference regarding powder ranking and reproducibility and was maintained explicitly throughout the analysis to avoid pseudoreplication. Accordingly, no factorial ANOVA based on specimen-level values was applied in the present study. 

The first level of analysis comprised descriptive statistics. Arithmetic means, standard deviations, and coefficients of variation were calculated where replicate specimen measurements were available. Potentially atypical observations were examined using robust deviations from the median and the median absolute deviation (MAD), with observations exceeding |robust z| > 3.5 flagged for further interpretation. Such observations were not automatically removed because an atypical response may reflect genuine incompatibility between a commercial powder and the concrete matrix rather than analytical error. Because each condition contained only six powders, MAD-based robust z-scores were treated as qualitative screening flags rather than precise inferential statistics; with *n* = 6, both the median and MAD were sensitive to the small sample configuration.

To enable comparison between the C12/15 and C20/25 concretes, the performance of each limestone-containing mixture was expressed relative to the corresponding class-specific reference concrete. The 7-day and 28-day compressive-strength retention ratios were calculated as follows (1):

(1) $$R_{7}=\frac{f_{c.7,i}}{f_{c,7,REF}},\;\;R_{28}=\frac{f_{c.28,i}}{f_{c,28,REF}}$$

where $f_{c.7,i}$ and $f_{c.28,i}$—mean compressive strengths of the limestone-containing mixture after 7 and 28 days and $f_{c,7,REF}$ and $f_{c,28,REF}$—corresponding mean compressive strengths of the class-specific reference concrete. 

Values of R7 or R28 equal to 1 indicate strength equal to that of the reference concrete; values greater than 1 indicate higher strength; and values lower than 1 indicate a reduction relative to the reference mixture.

For water penetration, an inverse performance ratio was adopted (2):

(2) $$R_{WP}=\frac{{WP}_{REF}}{{WP}_{i}}$$

where ${WP}_{i}$—mean maximum depth of water penetration for the limestone-containing concrete and ${WP}_{REF}$—corresponding value for the class-specific reference concrete. 

Slump and air content were expressed as relative deviations from the corresponding class-specific reference values. Positive values indicate an increase relative to the reference concrete, whereas negative values indicate a decrease. These deviations were treated as response descriptors rather than as inherently favourable or unfavourable performance indicators.

Formulation (2) was selected so that values greater than 1 consistently represent an improvement in water-penetration performance relative to the class-specific reference concrete, whereas values below 1 indicate increased penetration. Slump and air content were expressed as relative deviations from the corresponding reference values. This normalisation reduced baseline differences between the C12/15 and C20/25 concretes and enabled comparison on a common scale. However, each class-specific reference concrete was itself represented by a single batch. The normalised ratios therefore inherit uncertainty from both the limestone-containing batch and the reference batch; values close to 1.0, such as R28 = 1.094, should consequently be interpreted as approximate relative performance rather than proof of a reproducible improvement unless confirmed by independent batch replication. 

Relationships between the normalised performance indicators were evaluated using Spearman rank correlation. In addition, the stability of powder rankings between concrete matrices and replacement levels was assessed using rank-based comparisons. Because the 24 observations comprised repeated evaluations of the same six powders under four related conditions, they are not independent draws from a common population. Accordingly, pooled correlation coefficients and associated *p*-values were interpreted as exploratory descriptors of the response structure rather than confirmatory tests. To check whether the strongest pooled relationships were driven only by between-condition shifts, selected correlations were also recalculated separately within each of the four class-replacement subsets (*n* = 6 per subset). 

Principal component analysis (PCA) was then applied to the standardised, reference-normalised response matrix as an exploratory dimension-reduction method. Prior to PCA, each variable was centred and scaled to unit variance. Because the 24 rows represent six powders repeatedly evaluated under four systematic conditions, the PCA score structure may reflect both powder-specific effects and common shifts associated with concrete class or replacement level. The number of retained components was evaluated from explained variance and the scree plot, while component loadings were used to identify variables contributing most strongly to differentiation among the observed variants. 

Hierarchical cluster analysis was used as a complementary exploratory grouping method at the powder level. The four condition-specific response vectors for each powder were concatenated, producing a 6 × 20 powder–feature matrix. Each of the 20 features was standardised across the six powders; Euclidean distances were calculated; and Ward’s criterion was used for agglomeration. Because only six powders were available, clustering was not treated as a statistically validated classification. Two- and three-cluster cuts were examined explicitly; silhouette coefficients were calculated as descriptive support for the cut choice; and complete-linkage and average-linkage solutions were used as sensitivity checks of group membership. PCA and clustering were interpreted jointly but not as independent confirmatory evidence: PCA described variation among the 24 concrete variants, whereas clustering summarised similarities among six complete powder-response profiles.

The reference concretes were used exclusively as class-specific technological benchmarks and for normalisation. They were not treated as an additional “powder type” in PCA or clustering because they did not belong to the same material-factor structure as the limestone-containing variants. All multivariate analyses were performed on batch-level values. The adopted workflow therefore emphasised screening, response-pattern recognition, and exploratory grouping rather than formal inference based on artificial replication.

## 3. Results and Statistical Analysis

### 3.1. Overall Variability of Fresh- and Hardened-Concrete Properties

The experimental results showed a wide variation in the response of concretes containing the six commercial limestone powders, despite the fact that the mixture composition within each concrete class was kept constant apart from the fly-ash/limestone-powder substitution. In the C12/15 concrete with 50% fly-ash replacement, slump values ranged from 140 to 230 mm, air content from 3.0% to 6.0%, 7-day compressive strength from 9.43 to 12.87 MPa, 28-day compressive strength from 13.67 to 18.60 MPa, and mean water-penetration depth from 20.0 to 53.3 mm. At the 100% replacement level, the corresponding ranges were 170–230 mm, 4.2–6.5%, 8.27–12.00 MPa, 13.50–16.27 MPa, and 21.7–52.0 mm, respectively.

The C20/25 concrete displayed an equally broad material-dependent response. At 50% fly-ash replacement, slump varied from 200 to 250 mm, air content from 2.5% to 5.0%, 7-day compressive strength from 15.00 to 20.57 MPa, 28-day compressive strength from 21.27 to 30.53 MPa, and water penetration from 19.7 to 48.3 mm. At 100% replacement, the ranges were 190–250 mm, 2.3–5.8%, 11.03–18.90 MPa, 16.07–27.47 MPa, and 25.0–38.7 mm. These ranges demonstrate substantial variability in the concrete response associated with the different commercial limestone powders.

#### 3.1.1. Descriptive Statistics and Between-Powder Variability

The descriptive statistics calculated across the six powder variants within each concrete class–replacement combination are summarised in Table 3. 

The largest relative dispersion among the primary measured properties was generally observed for water penetration. In C12/15 concrete, the coefficient of variation for water penetration reached 36.0% at 50% replacement and 29.9% at 100% replacement, while in C20/25, the corresponding values were 30.8% and 15.5%. Air content also showed substantial between-powder variability, particularly in C20/25 concrete, where the coefficient of variation was 29.1% at 50% replacement and 28.0% at 100% replacement. By comparison, the coefficients of variation for 28-day compressive strength ranged from 7.3% to 16.6%. These results indicate that transport-related and fresh-concrete responses exhibited variability comparable to, or greater than, that observed for compressive strength, further supporting the use of a multivariable assessment rather than strength-only screening.

The coefficients of variation are compared graphically in Figure 2, highlighting the particularly high between-powder variability of water penetration and air content.

#### 3.1.2. Robust Screening of Atypical Responses

The within-batch dispersion of the three specimen results was evaluated separately from the between-mixture variability. Standard deviations and coefficients of variation were calculated for 7-day strength, 28-day strength, and water penetration. These values were used as indicators of measurement dispersion only and were not interpreted as independent experimental replication. The complete calculation table, including individual specimen results, arithmetic means, standard deviations, and coefficients of variation, is provided in Supplementary File S1.

Robust screening based on the median absolute deviation identified two observations above the adopted |robust z| > 3.5 flag, both for the normalised water-penetration indicator in C12/15 concrete containing Powder B. The 50% and 100% variants reached RWP values of 1.30 and 1.20, corresponding to nominal robust z-scores of 4.65 and 4.03, respectively. These observations were retained because they represented favourable, physically plausible performance rather than obvious measurement errors. However, because the MAD calculation for each condition was based on only six powders, the numerical z-scores should not be interpreted with high precision; their role is only to flag observations requiring substantive review.

Across all five normalised indicators, the two flagged observations were therefore highly specific rather than part of a general pattern of extreme values. For comparison, the largest non-flagged robust deviations were 3.281 for ΔAir/REF in A-100-C20/25, −2.777 for R7 in F-100-C20/25, and −2.444 for R28 in F-100-C20/25. This supports interpretation of the high RWP values for Powder B in C12/15 concrete as a distinctive matrix-specific response rather than as part of a general pattern of extreme observations.

### 3.2. Reference-Normalised Performance

Normalisation relative to the class-specific reference concretes enabled the response of the two concrete matrices to be compared on a common basis. For 28-day compressive strength, the mean retention ratio R28 for the six powders was 0.951 in C12/15 concrete at 50% fly-ash replacement and 0.866 at 100% replacement. In C20/25 concrete, the corresponding mean values were lower, at 0.812 and 0.719, respectively. Thus, increasing the fly-ash replacement level from 50% to 100% was associated with a general downward shift in the 28-day strength-retention ratio, particularly in the higher-strength C20/25 matrix.

The highest individual R28 values were obtained for Powder C in C12/15 concrete at 50% replacement (R28 = 1.094) and Powder D under the same conditions (R28 = 1.076). These values indicate that, in this matrix and at partial fly-ash replacement, the 28-day compressive strength slightly exceeded that of the reference concrete. Powder D also showed the highest overall mean R28 across the four investigated class–replacement combinations (0.960), followed by Powder C (0.926). By contrast, Powder F showed the lowest overall mean R28 (0.732), including a value of only 0.501 in C20/25 concrete at 100% fly-ash replacement. Figure 3 compares R28 for all six powders in the four experimental conditions.

As shown in Figure 4, the water-penetration ratio RWP revealed a different ranking of the materials. The mean RWP values for C12/15 concrete were 0.796 at 50% replacement and 0.768 at 100% replacement. In C20/25 concrete, the corresponding means were 0.486 and 0.473. Powder B was distinguished by the most favourable water-penetration response in C12/15 concrete, with RWP = 1.30 at 50% replacement and 1.20 at 100% replacement, indicating lower penetration depths than in the reference concrete. However, the same powder reached RWP values of only 0.430 and 0.506 in C20/25 concrete. This marked matrix-dependent response indicates that rankings obtained for one concrete composition may not be directly transferable to another.

#### 3.2.1. Complete Reference-Normalised Response Matrix

The complete reference-normalised response matrix is presented in Table 4. 

These five indicators constituted the common basis for the multivariate analyses. The 24 × 5 variant-level response matrix was used for correlation analysis and PCA, while the same indicators were subsequently reorganised into the 6 × 20 powder-level matrix used for hierarchical clustering. Across the 24 limestone-containing variants, ΔSlump/REF ranged from −0.300 to 0.250 and ΔAir/REF from −0.444 to 2.222. The strength-retention ratios ranged from 0.508 to 1.081 for R7 and from 0.501 to 1.094 for R28, while RWP ranged from 0.297 to 1.300. The most favourable individual 28-day strength response was recorded for C-50-C12/15 (R28 = 1.094), whereas the lowest R28 value occurred for F-100-C20/25 (0.501). The highest RWP value was obtained for B-50-C12/15 (1.300), while the lowest was observed for F-50-C20/25 (0.297). The response profiles were therefore clearly multidimensional: variants showing comparatively favourable strength retention did not necessarily exhibit the most favourable water-penetration response, while changes in slump and air content varied across concrete matrices and replacement levels.

#### 3.2.2. Standardised Multivariate Response Profiles

To visualise the variant-level multivariate structure used for PCA, the five reference-normalised indicators were centred and scaled to unit variance. The resulting standardised response profiles are shown in Table 5.

The heatmap makes the product- and matrix-specific contrasts directly visible: Powders C and D are associated with comparatively favourable strength-retention profiles in several conditions; Powder B displays a distinctly high water-penetration performance ratio in C12/15 concrete; and Powder F shows a pronounced negative mechanical profile in the C20/25 variants. This visual pattern is consistent with the subsequent PCA score structure and Ward grouping.

#### 3.2.3. Rank Stability Across Replacement Levels

The 50% to 100% replacement trajectories provided an additional measure of response stability. For 28-day strength, the rank ordering of the powders was relatively stable in C12/15 concrete, with Spearman ρ = 0.886 (*p* = 0.0188), and moderately stable in C20/25 concrete, with ρ = 0.771 (*p* = 0.0724). For water penetration, the corresponding rank correlations were ρ = 0.812 (*p* = 0.0499) for C12/15 and ρ = 0.522 (*p* = 0.288) for C20/25. These results indicate that powder rankings were more stable between the two replacement levels in the C12/15 concrete, whereas greater changes in relative ranking were observed in the C20/25 matrix.

### 3.3. Correlation Structure of the Performance Indicators

Spearman rank correlation analysis of the pooled 24-variant dataset revealed a strong positive relationship between the normalised 7-day and 28-day compressive-strength ratios (ρ = 0.839, *p* < 0.001). The direction of this relationship was preserved within each class-replacement subset: ρ ranged from 0.486 to 0.943 across the four six-powder subsets, although the small within-subset sample size limits formal significance testing. By contrast, the pooled associations involving RWP were less stable within individual conditions, supporting the interpretation that water-penetration behaviour is more strongly matrix-dependent. The pooled RWP correlations were ρ = 0.405 (*p* = 0.049) with R7 and ρ = 0.474 (*p* = 0.019) with R28. These results are therefore interpreted as exploratory structure rather than evidence from 24 independent samples.

Relative slump deviation was negatively correlated with both strength-retention indicators. The correlation coefficients were ρ = −0.777 (*p* < 0.001) for R7 and ρ = −0.689 (*p* < 0.001) for R28. Relative air-content deviation was also negatively related to R28 (ρ = −0.595, *p* = 0.002) and to RWP (ρ = −0.488, *p* = 0.016). These associations do not imply causality; however, they indicate systematic relationships among the measured response variables and show that changes in workability and air content accompanied changes in mechanical and transport-related performance. The complete Spearman correlation matrix is shown in Table 6.

### 3.4. Principal Component Analysis

#### 3.4.1. Explained Variance and Component Retention

PCA was performed on the five standardised reference-normalised indicators. The first principal component had an eigenvalue of 2.977 and explained 59.53% of the total variance. PC2 had an eigenvalue of 1.086 and explained a further 21.73%, giving a cumulative explained variance of 81.26% for the first two components. PC3 explained 11.42%, while PC4 and PC5 accounted for only 5.45% and 1.87%, respectively. The scree plot therefore supports focusing the interpretation primarily on the PC1–PC2 plane (Figure 5).

#### 3.4.2. Loading Structure

The loading structure provides a clear interpretation of the first two components. PC1 was dominated by R28 (loading = 0.950) and R7 (0.893), with an additional positive contribution from RWP (0.555), whereas relative slump deviation had a strong negative loading (−0.816). PC1 can therefore be interpreted primarily as a response axis dominated by strength retention, with an opposing contribution from relative slump deviation. The latter reflects the direction of covariation rather than an inherently favourable or unfavourable change in consistency. PC2 was characterised mainly by positive loading for relative air-content deviation (0.657) and negative loading for RWP (−0.620), with a smaller negative contribution from relative slump deviation (−0.401). PC2 therefore primarily represents variation in air-content and water-penetration response, with an additional contribution from consistency changes (Figure 6).

#### 3.4.3. Score-Space Interpretation

The PCA score plot (Figure 7) revealed clear differences among the 24 concrete variants, reflecting both powder-specific response patterns and changes associated with concrete class and fly-ash replacement level.

The most positive PC1 scores were observed for C-50-C12/15 (PC1 = 3.583) and D-50-C12/15 (PC1 = 2.970), consistent with their comparatively favourable strength-retention profiles. At the opposite end of PC1, F-100-C20/25 reached −3.644 and F-50-C20/25 reached −2.469, reflecting the relatively low R7 and R28 values recorded for Powder F in the C20/25 matrix. The substantial shifts in score position observed for the same powder across concrete classes and replacement levels indicate that the PCA plane captures a combination of powder-specific behaviour and systematic condition effects; it should therefore not be interpreted as if the 24 scores represented independent materials.

### 3.5. Stability of Powder Rankings Across Concrete Matrices

The comparison of powder rankings between the two concrete classes further indicated matrix-dependent behaviour. For R28, the rank correlation between C12/15 and C20/25 was ρ = 0.771 at 50% replacement and ρ = 0.543 at 100% replacement. The lower rank agreement at complete fly-ash replacement indicates greater divergence in powder rankings between the two concrete matrices at the higher replacement level. The corresponding R7 correlations were ρ = 0.600 and −0.429, respectively, indicating particularly low cross-matrix stability of the early-age-strength ranking at 100% replacement.

Water penetration showed the weakest cross-matrix agreement. At 50% replacement, the C12/15 and C20/25 RWP rankings showed very low agreement (ρ = −0.203), whereas at 100% replacement the correlation increased to ρ = 0.638, indicating a moderate degree of rank agreement. Given the small number of powders, these coefficients are interpreted descriptively as measures of ranking stability rather than as confirmatory statistical evidence. From a qualification perspective, this result is particularly relevant because it indicates that a powder performing favourably with respect to water penetration in one concrete class cannot automatically be assumed to behave similarly in another matrix.

### 3.6. Exploratory Hierarchical Grouping of Limestone Powders

#### 3.6.1. Cluster Formation and Fusion Distances

Hierarchical cluster analysis was performed at the powder level using the complete standardised response profiles across all four concrete class–replacement combinations. For each powder, the five normalised indicators from the four experimental conditions formed a 20-feature response vector. According to Ward’s agglomerative procedure, Powders D and E were merged first at a fusion distance of 4.210, followed by Powders A and B at a distance of 4.604. Powder C subsequently joined the D–E cluster at a distance of 4.794, whereas Powder F merged with the A–B branch only at a considerably larger distance of 7.211. The final merger between the two major branches, i.e., {C, D, E} and {A, B, F}, occurred at a Ward distance of 9.279.

#### 3.6.2. Descriptive Grouping and Cluster-Solution Sensitivity 

The two-cluster cut separated the powders into C–D–E and A–B–F. At the three-cluster cut, Powder F formed a separate subgroup, while A–B and C–D–E remained grouped. Because the analysis contains only six objects, neither cut is presented as a uniquely validated functional classification. The silhouette coefficient was 0.237 for two clusters and 0.255 for three clusters, indicating only a modest descriptive preference for the three-cluster solution. Importantly, both complete-linkage and average-linkage sensitivity analyses reproduced the same two-cluster partition and the same three-cluster structure (A–B, C–D–E, F). The grouping is therefore reasonably stable to the linkage rule within this small dataset, but it is used only as a descriptive aid for comparing complete response profiles. Powders C and D were associated with relatively high strength retention under several conditions, whereas Powder F showed the strongest deterioration in C20/25 concrete, particularly at complete fly-ash replacement. Powder B remained distinctive because of its favourable C12/15 water-penetration response despite joining A and F in the two-cluster partition with coarser materials.

The Ward agglomeration sequence and fusion distances are shown as a conventional dendrogram in Figure 8. The two- and three-cluster interpretations discussed above are alternative descriptive cuts of the same hierarchy; the small difference in silhouette coefficients does not justify treating either cut as a definitive material taxonomy.

### 3.7. Integrated Performance Interpretation

The combined descriptive, normalised, correlation, PCA, ranking, and clustering results indicate that no single limestone powder exhibited consistently superior performance across all concrete matrices and replacement levels. Powders C and D provided the highest overall 28-day strength retention, with mean R28 values of 0.926 and 0.960, respectively, across the four investigated conditions. Powder B showed the most favourable water-penetration response in C12/15 concrete but did not retain this advantage in C20/25 concrete. Powder F exhibited the lowest overall mean R28 (0.732) and the most negative PCA scores in the C20/25 variants, indicating a less favourable mechanical response under those conditions.

The multivariate analyses therefore support the central premise of the study that nominally similar commercial limestone powders may exhibit substantially different functional responses. Their performance varied with both powder identity and concrete environment. Consequently, qualification based on a single property or replacement level would provide an incomplete basis for material selection. The combined use of reference-normalised indicators, rank-stability analysis, PCA, and hierarchical clustering provided a broader screening framework capable of identifying overall response trends as well as matrix-dependent changes.

Taken together, the descriptive variability, robust screening, standardised response profiles, and multivariate analyses consistently indicate that powder identity is relevant to the observed concrete response. The following Discussion section therefore focuses on the implications of this functional variability for material qualification, the role of the concrete matrix, and the limitations of transferring performance rankings between mixture conditions.

## 4. Discussion

### 4.1. Functional Variability of Commercial Limestone Powders

The results show that commercial limestone powders belonging to the same broad material category may produce markedly different concrete responses, even when used at the same mass replacement level and with the remaining mixture constituents kept unchanged. Similar variability has been reported in the literature and has been linked to differences in fineness, particle-size distribution, morphology, mineralogical composition, and interactions with the cementitious system [12,13,14,15,16,17,18,19,20,21,22]. As shown in Table 1, the powders investigated in this study also differed in selected chemical, mineralogical, and physical characteristics. These data provide useful material context. They were not included in the primary multivariate screening based on concrete-performance responses, but were subsequently examined in a separate exploratory powder-level correlation analysis. Given the small number of powders, no direct causal relationship between individual material characteristics and concrete performance is inferred here. The findings nevertheless show that variability between market-available limestone powders remains relevant when their performance is assessed directly in concrete.

The differences were particularly evident in the normalised 28-day strength and water-penetration indicators. Powders C and D achieved the highest overall strength-retention ratios, while Powder F exhibited a markedly less favourable mechanical response in the C20/25 matrix. By contrast, Powder B showed an unusually favourable water-penetration response in C12/15 concrete but did not retain this advantage in C20/25. These contrasting trends indicate that assessment based on a single performance indicator would be insufficient. A powder that performs well in terms of compressive strength is not necessarily the best-performing material with respect to transport-related behaviour, and vice versa.

An exploratory powder-level comparison was also made between the material descriptors in Table 1 and the mean performance ratios across the four concrete conditions. With only six powders, no single descriptor showed a strong monotonic relationship with mean R28 or mean RWP. Spearman correlations with mean R28 were ρ = 0.143 for CaO content, ρ = −0.086 for Blaine fineness, ρ = 0.143 for calcite content, and ρ = −0.029 for d50. The corresponding correlations with mean RWP were ρ = 0.086, 0.143, 0.086, and −0.314, respectively. Given the very small number of powders, these coefficients are interpreted descriptively and should not be used for mechanistic or causal attribution. Nevertheless, the weak relationships indicate that the observed performance differences cannot be explained by any single material descriptor alone and support the need for multivariable, matrix-specific qualification.

This finding agrees with previous studies reporting that limestone can simultaneously influence packing, hydration kinetics, dilution, and pore structure [12,13,14,15,16,17,18]. Depending on the balance among these mechanisms, improvements in one response may coexist with deterioration in another. In practical terms, this means that a limestone powder should not be qualified solely by its commercial designation or by one isolated laboratory property. Instead, its suitability should be verified in the target concrete matrix using several complementary performance indicators.

### 4.2. Influence of Concrete Matrix and Fly-Ash Replacement Level

The comparison between C12/15 and C20/25 concretes revealed clear differences in the relative ranking of the powders. Overall, the reference-normalised strength and water-penetration responses were more favourable and more stable in C12/15 than in C20/25. The lower agreement between the rankings obtained for the two concrete classes, particularly at 100% fly-ash replacement, shows that the performance of a given powder cannot be separated from the composition of the concrete in which it is used.

The replacement level also affected the response. Partial substitution of fly ash by limestone powder at 50% generally resulted in higher 28-day strength-retention ratios than complete substitution at 100%. This trend agrees with the complementary behaviour of fly ash and limestone reported in previous studies. Fly ash contributes through delayed pozzolanic reaction, whereas limestone can affect particle packing, nucleation, and carbonate–aluminate interactions [16,17,18,32]. Retaining part of the fly ash therefore preserves a reactive aluminosilicate fraction, while limestone may contribute mainly through physical and early-age effects. When fly ash is completely replaced, the amount of reactive supplementary material is reduced and dilution effects may become more pronounced [12,13,17,18].

The wider variation observed in the C20/25 concrete further illustrates that powder response depends on the surrounding matrix. A further consideration is that fly ash was replaced by limestone on a mass basis while the powders differed slightly in density and markedly in fineness; because detailed fly-ash density and fineness were unavailable in the archived dataset, associated changes in solid volume and packing cannot be quantified separately. The present results therefore support matrix-specific verification but do not isolate the individual physicochemical mechanisms responsible for the observed differences.

### 4.3. Interpretation of the Multivariate Analysis

The correlation, PCA, and hierarchical clustering results provided complementary views of the relationships among the measured concrete responses. The strong correlation between R7 and R28 shows that mixtures with relatively high early-age strength retention generally maintained this tendency at 28 days. The weaker relationships between the strength indicators and RWP, however, show that water-penetration behaviour contributed additional information that could not be inferred from compressive strength alone.

PCA explained 81.26% of the response variance in the first two components and was useful for visualising the combined mechanical, transport, and fresh-concrete response structure. Hierarchical clustering provided a descriptive grouping of complete powder profiles; the same two- and three-cluster memberships were reproduced by alternative linkage methods, but the small six-powder sample precludes treating these groups as a validated functional taxonomy.

Ward clustering offered a complementary powder-level perspective, but its role is descriptive because only six powders were grouped. The C–D–E versus A–B–F split was preserved under complete and average linkage, and the three-cluster cut consistently isolated Powder F. The slightly higher silhouette coefficient for three clusters (0.255 versus 0.237 for two clusters) indicates that separating F is plausible, but the difference is too small to justify a definitive taxonomy. The dendrogram should therefore be read as a compact visual summary of response-profile similarity rather than as a formal functional classification.

From an engineering perspective, the main value of the multivariate approach lies in screening and material qualification rather than in identifying one universally superior powder. PCA helps to identify which measured responses contribute most strongly to differentiation between concrete variants, while hierarchical clustering reveals similarities among the overall powder response profiles. Together with reference-normalised indicators and ranking-stability analysis, these methods provide a practical framework for identifying consistent response patterns, matrix-sensitive behaviour, and atypical material responses. 

### 4.4. Practical Implications and Limitations

The results have practical implications for the use of commercially available limestone powders as fly-ash replacements. Product-specific verification is particularly important when complete fly-ash replacement is considered, and it should be carried out in the intended concrete matrix using several relevant properties. For preliminary engineering screening, a provisional ±10% band around the class-specific reference value (0.90–1.10) may be used as a practical trigger for the normalised R28 and RWP indicators: values outside this band would indicate that interchangeability should not be assumed without further testing. Conversely, values within this band should not be interpreted as proof of interchangeability, particularly in the absence of independent batch replication. This ±10% band is proposed only as an illustrative screening tolerance, not as a validated acceptance criterion; project-specific strength, durability, consistency, air-content, and specification requirements remain controlling.

Several limitations should be considered when interpreting the findings. Each powder–class–replacement combination was represented by a single laboratory batch, so specimen-level strength and water-penetration measurements quantify only within-batch test dispersion and do not capture batch-to-batch variability associated with mixing day, aggregate moisture, or production conditions. The 24 observations used in pooled correlation and PCA are repeated evaluations of six powders across four systematically different conditions rather than 24 independent material samples. The class-specific reference concretes were also single batches, so normalised ratios inherit uncertainty from the reference value. In addition, the MAD screening groups contained only six powders, and the hierarchical clustering grouped only six objects. These features limit the precision of inferential *p*-values, outlier scores, rankings, and cluster interpretation. Finally, the available fly-ash characterisation was incomplete, preventing explicit separation of mass-replacement effects from differences in solid volume, fineness, and packing.

Further research should combine this screening framework with independent technological batch replication and a factorial Design of Experiments in which powder identity, concrete matrix, and replacement level can be evaluated with formal main-effect and interaction tests. Replicated batches would permit separation of material effects from day-to-day production variability and would support confidence intervals for powder rankings. More complete characterisation of both limestone powders and fly ash, including particle-size distribution, specific surface area, mineralogy, morphology, density, and water demand, should be incorporated as explanatory variables. Such work could also test and calibrate practical interchangeability tolerances rather than relying on an illustrative screening band, and could validate whether the descriptive PCA and clustering patterns observed here remain stable in independent datasets.

## 5. Conclusions

This study evaluated six commercial limestone powders used as 50% and 100% mass replacements of fly ash in C12/15 and C20/25 concretes. The conclusions apply only to the investigated concrete compositions, replacement levels, curing ages, and measured properties, and the analysis should be interpreted as exploratory performance screening because each experimental condition was represented by one laboratory batch:

- The powders produced markedly different fresh-concrete, strength, and water-penetration responses under otherwise comparable mixture conditions. Within the investigated matrices, this variability is sufficient to show that a common commercial designation alone is not an adequate basis for assuming functional interchangeability.
- Partial fly-ash replacement generally preserved 28-day strength more effectively than complete replacement: mean R28 decreased from 0.951 to 0.866 in C12/15 and from 0.812 to 0.719 in C20/25 when replacement increased from 50% to 100%.
- No powder was superior for every response. Powders C and D showed the highest mean 28-day strength retention, whereas Powder B was distinguished by favourable water-penetration performance only in C12/15 concrete; Powder F showed the least favourable overall strength retention. This supports the need for multi-property rather than strength-only screening.
- Powder rankings changed with concrete class and replacement level. Performance observed in one concrete matrix should therefore not be transferred to another without verification.
- PCA reduced the five-dimensional response space to two principal components explaining 81.26% of the total variance. PC1 was dominated by 7-day and 28-day strength retention, with an opposing contribution from relative slump deviation, while PC2 mainly reflected air-content variation and water-penetration response. Ward clustering revealed a descriptive two-group structure, C–D–E and A–B–F, with Powder F forming a separate subgroup in the three-cluster interpretation. These results highlight distinct multivariable response profiles among the investigated powders.
- The combined analytical approach provides an exploratory framework for product- and matrix-specific screening, but the principal limitation is the absence of independent batch replication. Future replicated DoE studies, coupled with complete material characterisation, are required to quantify powder, matrix, and replacement-level effects and to calibrate practical interchangeability criteria.

Overall, the suitability of a commercial limestone powder as a fly-ash replacement should be verified in the target concrete matrix using several complementary performance indicators and independent batch replication before broader qualification or substitution is recommended.

## Figures and Tables

**Figure 1:**
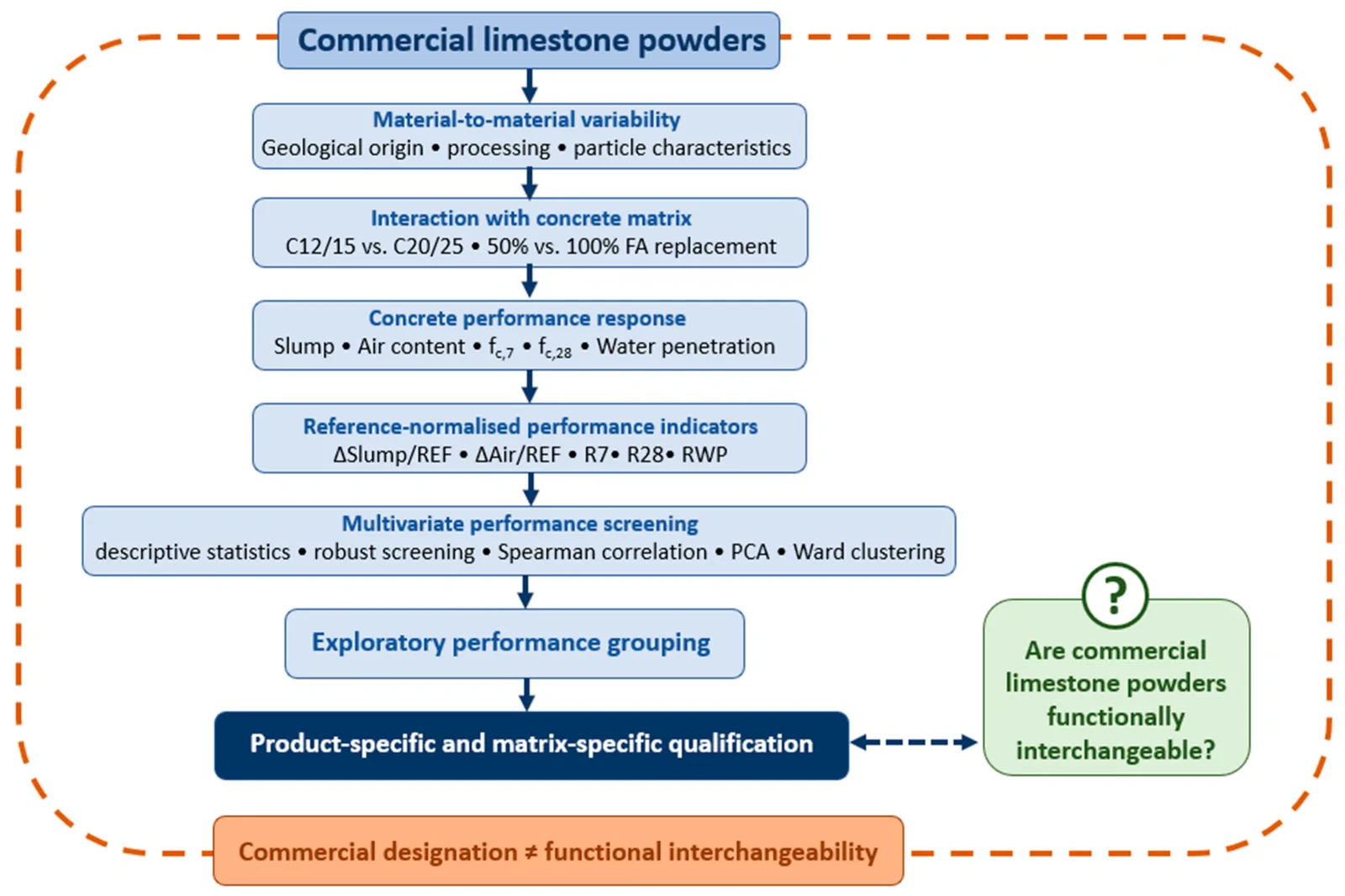
Conceptual framework linking commercial limestone-powder variability, concrete matrix effects, measured performance indicators, and multivariate performance-based screening.

**Figure 2:**
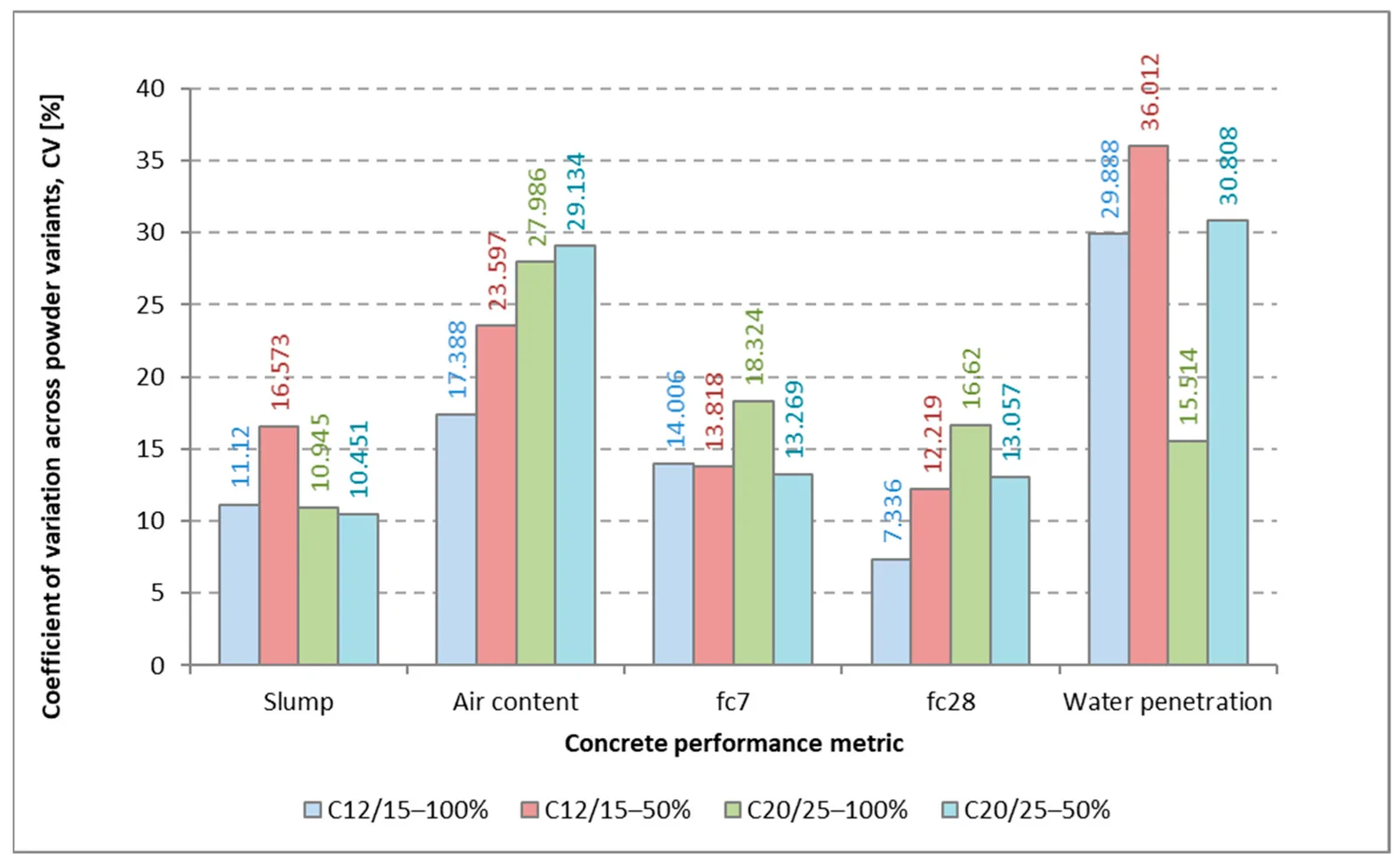
Coefficients of variation in the primary concrete properties across the six limestone-powder variants for the four concrete class–replacement combinations.

**Figure 3:**
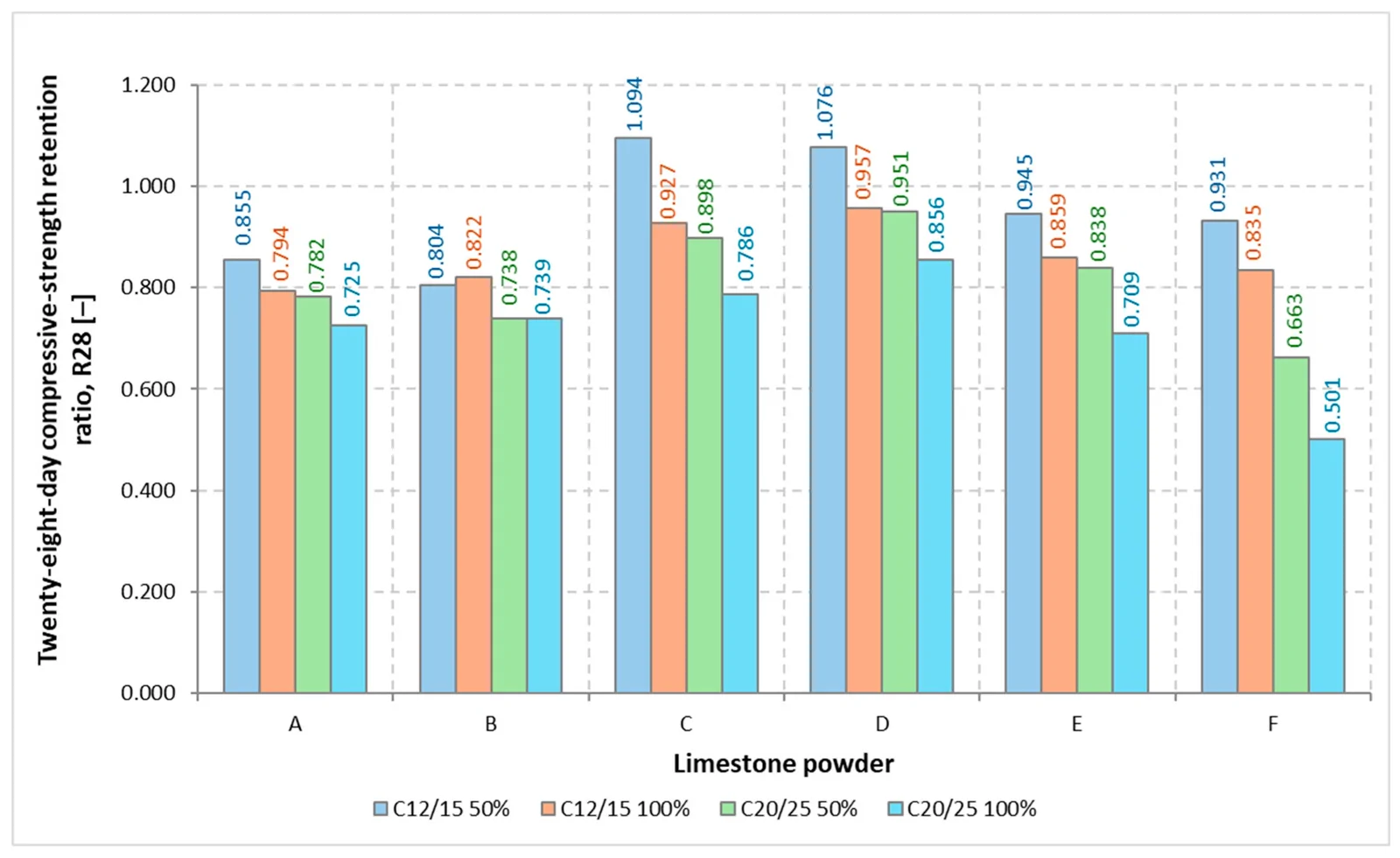
Twenty-eight-day compressive-strength retention ratio, R28, for the six limestone powders in C12/15 and C20/25 concretes at 50% and 100% fly-ash replacement.

**Figure 4:**
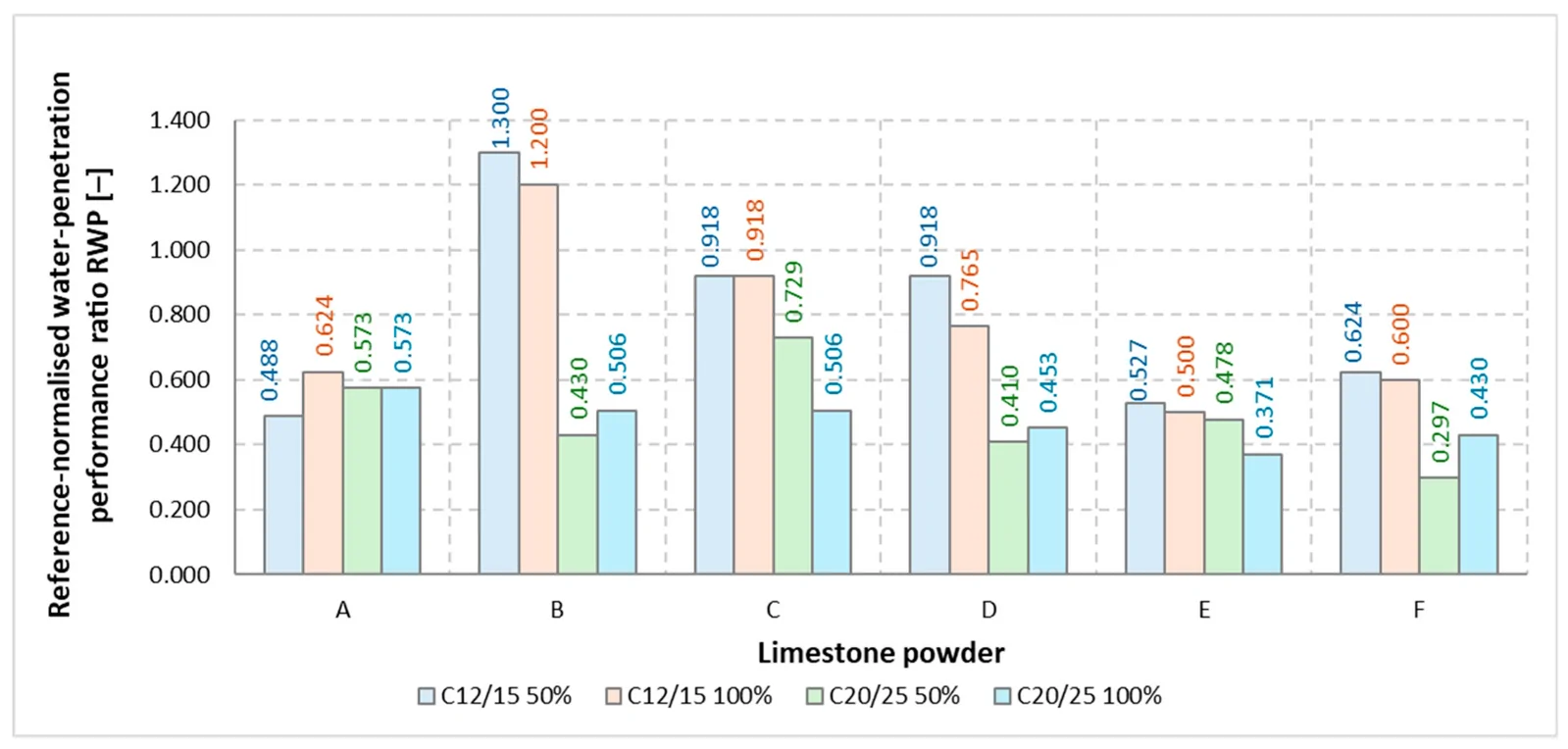
Reference-normalised water-penetration performance ratio, RWP, for the six limestone powders in the four concrete class–replacement combinations.

**Figure 5:**
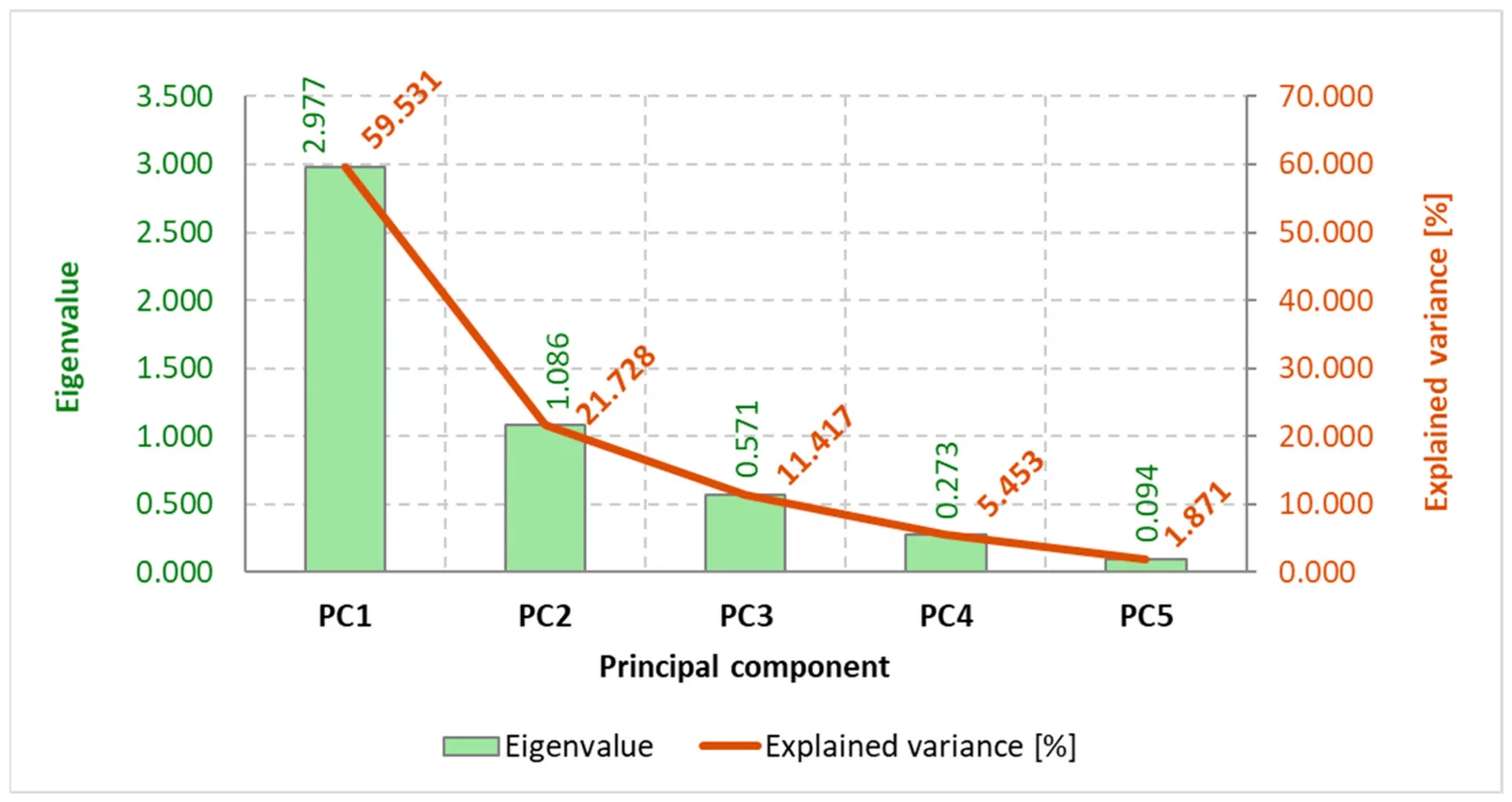
PCA scree plot showing the variance explained by the five principal components.

**Figure 6:**
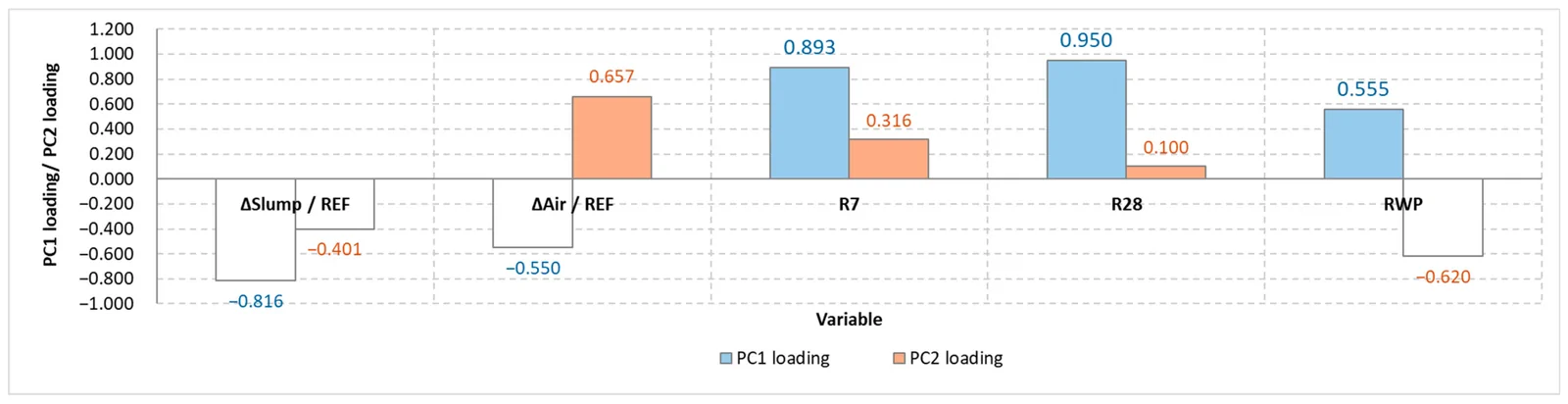
PCA loadings of the reference-normalised performance indicators on PC1 and PC2, showing the contribution and direction of each variable to the first two principal components.

**Figure 7:**
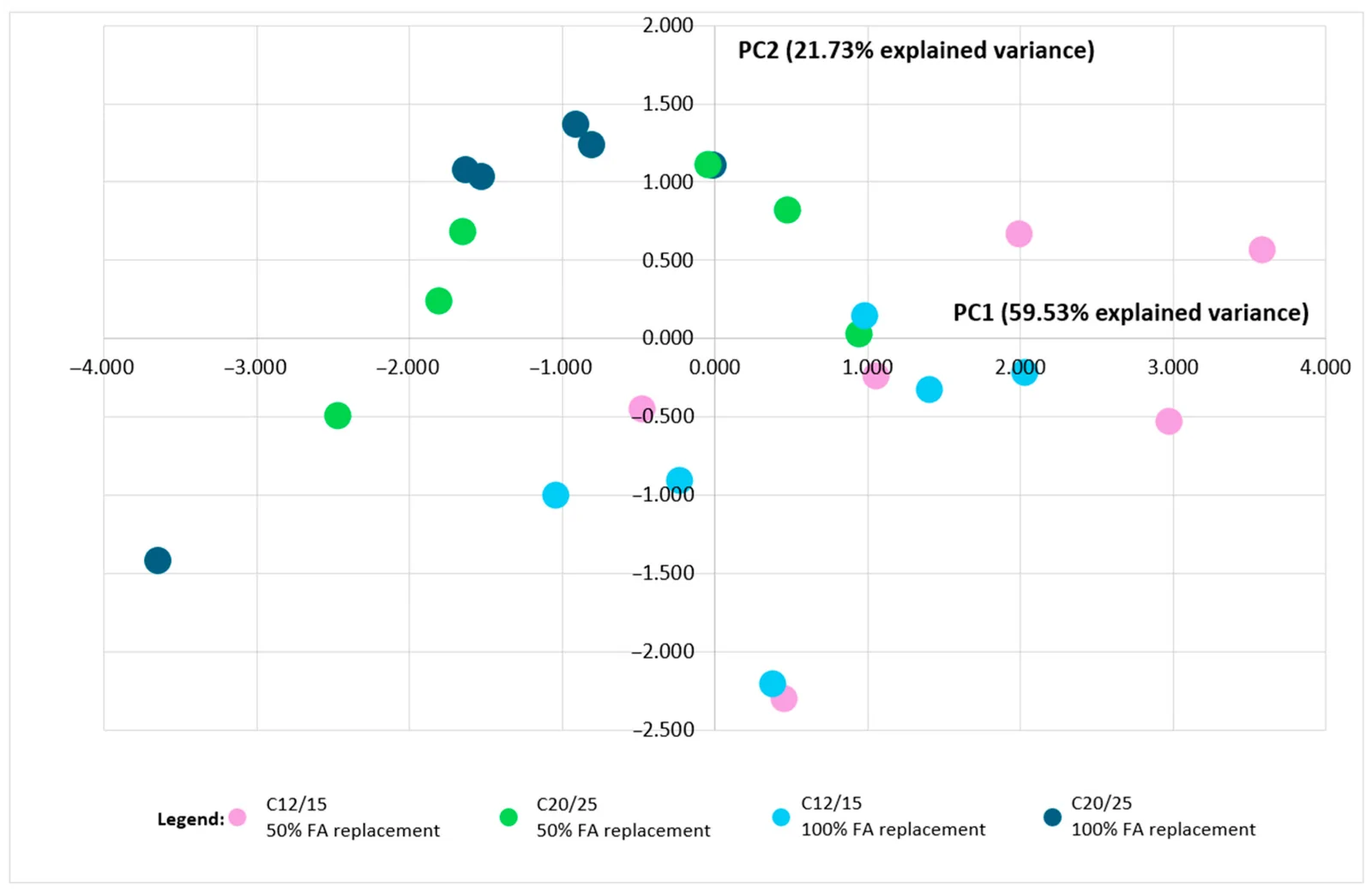
PCA score plot for the 24 limestone-containing concrete variants in the PC1–PC2 plane.

**Figure 8:**
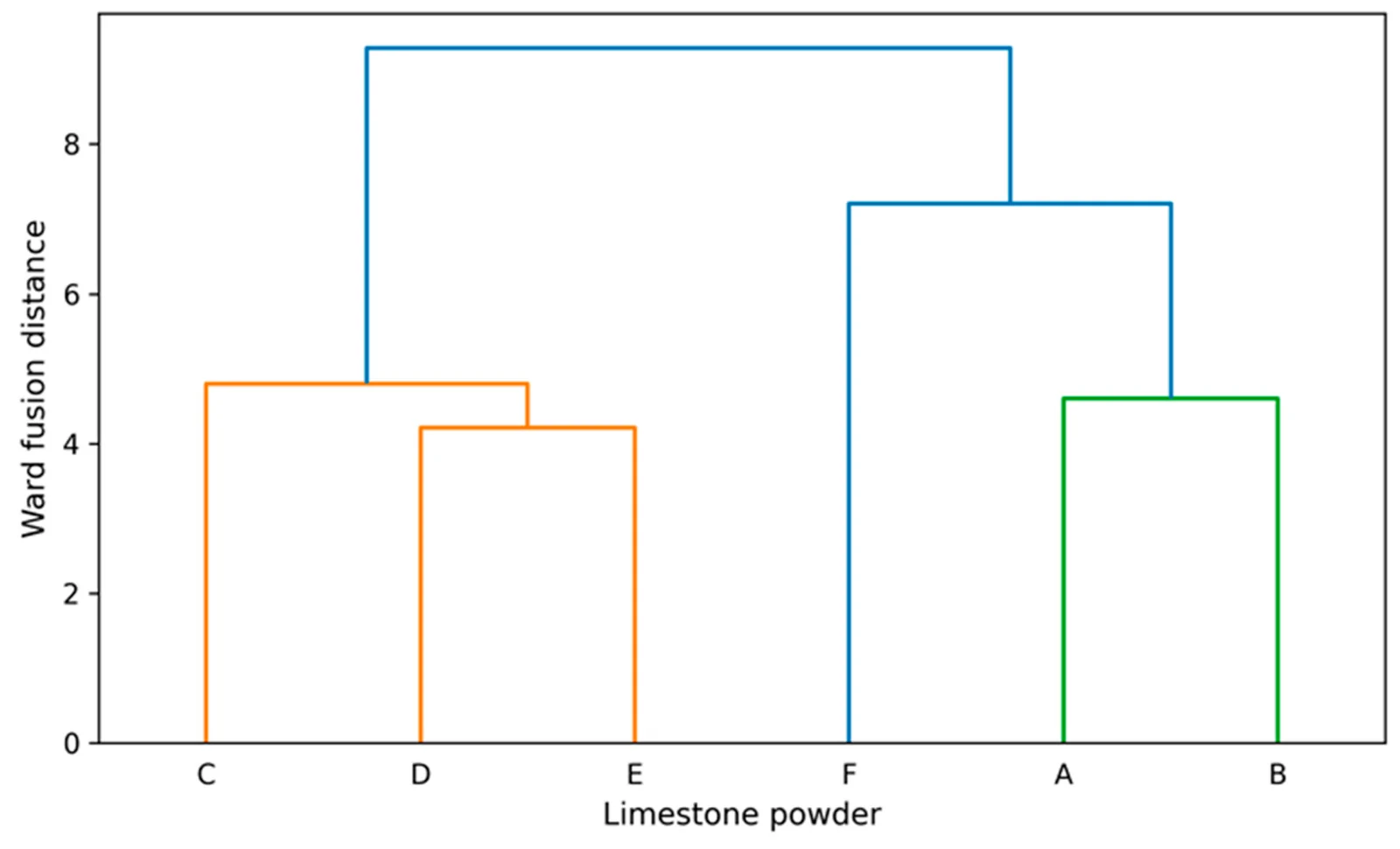
Ward hierarchical-clustering dendrogram for the six limestone powders based on standardised 20-feature response profiles across the four concrete conditions. Orange branches connect powders C–D–E, green branches connect A–B, and the blue branch leads to powder F and shows its subsequent merging with A–B and then C–D–E.

**Table 1:** Basic chemical, mineralogical, and physical characteristics of the investigated commercial limestone powders.

Parameter	Powder A	Powder B	Powder C	Powder D	Powder E	Powder F
CaO [%]	86.20	87.90	95.70	78.20	90.20	80.60
MgO [%]	2.80	2.20	0.50	8.51	1.80	5.60
SiO_2_ [%]	7.40	6.80	2.96	10.31	5.70	12.60
Loss on ignition [%]	41.30	41.60	42.69	41.11	41.90	40.20
Calcite, XRD [%]	88.00	90.20	98.59	71.09	93.20	78.50
Blaine-specific surface area [cm^2^/g]	4100	4550	4396	3804	5050	3150
Particle size, d_50_ [µm]	18.50	14.80	14.12	22.15	12.20	28.50
Density [g/cm^3^]	2.74	2.75	2.76	2.74	2.75	2.73

**Table 2:** Composition of reference and limestone-powder-modified concretes, kg/m^3^.

Component [kg/m^3^]	C12/15			C20/25		
	REF	50% FA Replacement	100% FA Replacement	REF	50% FA Replacement	100% FA Replacement
CEM I 42.5 R	210	210	210	280	280	280
Fly ash	100	50	0	100	50	0
Limestone powder	0	50	100	0	50	100
Water	220	220	220	225	225	225
Aggregate 0/2 mm	720	720	720	655	655	655
Aggregate 2/8 mm	400	400	400	400	400	400
Aggregate 8/16 mm	700	700	700	700	700	700
Plast 317	2.1	2.1	2.1	2.8	2.8	2.8

**Table 3:** Descriptive statistics of the primary concrete responses across the six limestone-powder variants.

Concrete Class	FA Repl. [%]	Metric	Mean	SD	Min	Max	Median	CV [%]
C12/15	50	Slump [mm]	193.333	32.042	140.000	230.000	195.000	16.573
C12/15	50	Air [%]	5.000	1.180	3.000	6.000	5.200	23.597
C12/15	50	fc7 [MPa]	11.339	1.567	9.433	12.867	11.617	13.818
C12/15	50	fc28 [MPa]	16.167	1.975	13.667	18.600	15.950	12.219
C12/15	50	Water penetration [mm]	36.833	13.264	20.000	53.333	35.000	36.012
C12/15	100	Slump [mm]	208.333	23.166	170.000	230.000	210.000	11.120
C12/15	100	Air [%]	5.267	0.916	4.200	6.500	5.450	17.388
C12/15	100	fc7 [MPa]	10.333	1.447	8.267	12.000	10.550	14.006
C12/15	100	fc28 [MPa]	14.717	1.080	13.500	16.267	14.400	7.336
C12/15	100	Water penetration [mm]	36.833	11.009	21.667	52.000	37.833	29.888
C20/25	50	Slump [mm]	221.667	23.166	200.000	250.000	215.000	10.451
C20/25	50	Air [%]	3.600	1.049	2.500	5.000	3.500	29.134
C20/25	50	fc7 [MPa]	18.194	2.414	15.000	20.567	18.467	13.269
C20/25	50	fc28 [MPa]	26.056	3.402	21.267	30.533	26.000	13.057
C20/25	50	Water penetration [mm]	31.889	9.824	19.667	48.333	31.667	30.808
C20/25	100	Slump [mm]	211.667	23.166	190.000	250.000	200.000	10.945
C20/25	100	Air [%]	4.450	1.245	2.300	5.800	4.800	27.986
C20/25	100	fc7 [MPa]	16.789	3.076	11.033	18.900	18.267	18.324
C20/25	100	fc28 [MPa]	23.089	3.837	16.067	27.467	23.500	16.620
C20/25	100	Water penetration [mm]	30.889	4.792	25.000	38.667	30.000	15.514

**Table 4:** Reference-normalised performance indicators used in the multivariate analysis.

Variant	ΔSlump/REF	ΔAir/REF	R7	R28	RWP
A-50-C12/15	0.150	0.111	0.812	0.855	0.488
A-50-C20/25	0.250	1.778	0.783	0.782	0.573
A-100-C12/15	0.150	0.204	0.695	0.794	0.624
A-100-C20/25	0.150	2.222	0.871	0.725	0.573
B-50-C12/15	0.100	0.074	0.793	0.804	1.300
B-50-C20/25	0.100	1.500	0.748	0.738	0.430
B-100-C12/15	0.150	−0.019	0.824	0.822	1.200
B-100-C20/25	0.000	1.889	0.862	0.739	0.506
C-50-C12/15	−0.300	0.111	1.039	1.094	0.918
C-50-C20/25	0.000	0.444	0.948	0.898	0.729
C-100-C12/15	−0.150	0.074	0.955	0.927	0.918
C-100-C20/25	0.000	1.778	0.826	0.786	0.506
D-50-C12/15	−0.050	−0.444	1.078	1.076	0.918
D-50-C20/25	0.050	0.667	0.942	0.951	0.410
D-100-C12/15	0.000	0.037	0.950	0.957	0.765
D-100-C20/25	−0.050	1.111	0.857	0.856	0.453
E-50-C12/15	−0.100	−0.148	1.081	0.945	0.527
E-50-C20/25	0.000	1.222	0.919	0.838	0.478
E-100-C12/15	0.000	−0.222	1.008	0.859	0.500
E-100-C20/25	0.000	1.556	0.717	0.709	0.371
F-50-C12/15	0.000	−0.148	0.913	0.931	0.624
F-50-C20/25	0.250	0.389	0.691	0.663	0.297
F-100-C12/15	0.100	−0.222	0.779	0.835	0.600
F-100-C20/25	0.250	0.278	0.508	0.501	0.430

**Table 5:** Heatmap-style representation of standardised reference-normalised performance indicators for the 24 limestone-containing concrete variants.

Variant	ΔSlump/REF	ΔAir/REF	R7	R28	RWP
A-50-C12/15	0.814	−0.596	−0.341	0.140	−0.559
A-50-C20/25	1.581	1.464	−0.555	−0.424	−0.226
A-100-C12/15	0.814	−0.481	−1.205	−0.332	−0.026
A-100-C20/25	0.814	2.012	0.094	−0.865	−0.226
B-50-C12/15	0.431	−0.642	−0.482	−0.254	2.623
B-50-C20/25	0.431	1.120	−0.814	−0.765	−0.787
B-100-C12/15	0.814	−0.757	−0.253	−0.115	2.231
B-100-C20/25	−0.335	1.601	0.027	−0.757	−0.489
C-50-C12/15	−2.635	−0.596	1.333	1.989	1.126
C-50-C20/25	−0.335	−0.185	0.662	0.473	0.385
C-100-C12/15	−1.485	−0.642	0.713	0.697	1.126
C-100-C20/25	−0.335	1.464	−0.238	−0.394	−0.489
D-50-C12/15	−0.719	−1.282	1.621	1.850	1.126
D-50-C20/25	0.048	0.091	0.617	0.883	−0.865
D-100-C12/15	−0.335	−0.688	0.677	0.929	0.526
D-100-C20/25	−0.719	0.640	−0.010	0.148	−0.697
E-50-C12/15	−1.102	−0.916	1.643	0.836	−0.407
E-50-C20/25	−0.335	0.777	0.448	0.009	−0.599
E-100-C12/15	−0.335	−1.008	1.104	0.171	−0.512
E-100-C20/25	−0.335	1.189	−1.042	−0.989	−1.018
F-50-C12/15	−0.335	−0.916	0.404	0.728	−0.026
F-50-C20/25	1.581	−0.253	−1.234	−1.345	−1.308
F-100-C12/15	0.431	−1.008	−0.585	−0.015	−0.121
F-100-C20/25	1.581	−0.390	−2.584	−2.598	−0.787

*Note: Cell background colours represent the magnitude of the standardised values: red/orange indicates lower values, yellow intermediate values, and green higher values.*

**Table 6:** Spearman rank correlation matrix for the reference-normalised performance indicators.

Variable	ΔSlump/REF	ΔAir/REF	R7	R28	RWP
ΔSlump/REF	1.000	0.244	−0.777	−0.689	−0.255
ΔAir/REF	0.244	1.000	−0.365	−0.595	−0.488
R7	−0.777	−0.365	1.000	0.839	0.405
R28	−0.689	−0.595	0.839	1.000	0.474
RWP	−0.255	−0.488	0.405	0.474	1.000

*Note: Cell background colours indicate the direction and strength of the correlations: green denotes positive correlations and red denotes negative correlations; stronger shading indicates a larger absolute correlation coefficient.*

## Data Availability

The data supporting the results presented in this study, including Supplementary File S1, are openly available in the GitHub repository at https://github.com/izas-prz/limestone-powders-concrete-supplementary-data.

## Supplementary Materials

The following supporting information can be downloaded at https://github.com/izas-prz/limestone-powders-concrete-supplementary-data (accessed on 23 September 2026). Supplementary File S1: The supplementary calculation workbook.

## Abbreviations

The following abbreviations are used in this manuscript:

SCM	Supplementary cementitious material
FA	Fly ash
REF	Reference concrete
PCA	Principal component analysis
PC	Principal component
MAD	Median absolute deviation
SD	Standard deviation
CV	Coefficient of variation
R7	7-day compressive-strength retention ratio
R28	28-day compressive-strength retention ratio
RWP	Reference-normalised water-penetration performance ratio
XRF	X-ray fluorescence
XRD	X-ray diffraction
